# H3K36 Di-Methylation Marks, Mediated by Ash1 in Complex with Caf1-55 and MRG15, Are Required during *Drosophila* Heart Development

**DOI:** 10.3390/jcdd10070307

**Published:** 2023-07-18

**Authors:** Jun-yi Zhu, Chen Liu, Xiaohu Huang, Joyce van de Leemput, Hangnoh Lee, Zhe Han

**Affiliations:** 1 Center for Precision Disease Modeling, Department of Medicine, University of Maryland School of Medicine, Baltimore, MD 21201, USA; 2 Division of Endocrinology, Diabetes, and Nutrition, Department of Medicine, University of Maryland School of Medicine, Baltimore, MD 21201, USA

**Keywords:** Ash1, Caf1-55, MRG15, Set2, H3K36, histone lysine methyltransferase, cardiac development, heart function, epigenetic regulation

## Abstract

Methyltransferases regulate transcriptome dynamics during development and aging, as well as in disease. Various methyltransferases have been linked to heart disease, through disrupted expression and activity, and genetic variants associated with congenital heart disease. However, in vivo functional data for many of the methyltransferases in the context of the heart are limited. Here, we used the *Drosophila* model system to investigate different histone 3 lysine 36 (H3K36) methyltransferases for their role in heart development. The data show that *Drosophila* Ash1 is the functional homolog of human ASH1L in the heart. Both Ash1 and Set2 H3K36 methyltransferases are required for heart structure and function during development. Furthermore, Ash1-mediated H3K36 methylation (H3K36me2) is essential for healthy heart function, which depends on both Ash1-complex components, Caf1-55 and MRG15, together. These findings provide in vivo functional data for Ash1 and its complex, and Set2, in the context of H3K36 methylation in the heart, and support a role for their mammalian homologs, ASH1L with RBBP4 and MORF4L1, and SETD2, during heart development and disease.

## 1. Introduction

Histone methylation provides a layer of regulatory oversight during the complex transitional landscapes that cover development, aging, and disease [1,2]. The methylation marks are both stable, when heritable, and dynamic, such as in response to an internal or environmental trigger. The marks on histone tails are dependent on the sequence, order, and prior processes, and they can be added and removed via numerous highly specialized enzymes; all this intricately connects them to a vast molecular-signaling network [3,4]. These characteristics have made histone methylation a crucial regulatory element during cardiac development, and its dysregulation has been associated with disease [3,5,6,7,8,9]. In fact, histone methyltransferases (KMTs) are enriched among genes with variants associated with congenital heart disease [10,11,12].

The histone 3 (H3) tail contains multiple lysine (K) methylation sites. Among these, methylation at H3K36 is highly conserved from fly to human. In fact, the SET domain present in all H3K36 methyltransferases was first discovered as a conserved sequence in three *Drosophila* proteins—suppressor of variegation 3-9 (Su(var)3-9), enhancer of zeste (E(z)), and the trithorax-group chromatin regulator, trithorax (Trx) [13,14,15]. This SET domain catalyzes the transfer of a methyl group from an S-adenosylmethionine donor molecule to histones or other proteins, with mono- (me1), di- (me2) and tri-methylation (me3) states at specific lysines catalyzed by specialized enzymes [16,17]. SET2 family members include nuclear-receptor-binding SET domain proteins, (NSD)1, NSD2, and NSD3, as well as ASH1-like histone lysine methyltransferase (ASH1L), which exert H3K36me1 and H3K36me2 activity (NSD3 H3K36me2) [18,19,20,21,22,23]. In addition, an SET domain containing 2, histone lysine methyltransferase (SETD2) exerts H3K36me3 activity [24]. H3K36 marks are generally associated with active chromatin, i.e., gene transcription [16,17].

Variants in the genes that encode the H3K36 methyltransferases, NSD1, NSD2, and, ASH1L, have been linked to congenital heart disease [11,12,25,26,27,28,29,30,31]. Unfortunately, few of the many genes that carry candidate variants for congenital heart disease have been validated in animal models [10,32]. For *NSD2*, its conditional knockdown in the myocardium of a mouse model for cardiac hypertrophy has demonstrated its role in ventricular remodeling linked to H3K36me2 [33]. For *Setd2*, its knockdown in mouse cardiac progenitors has disrupted cardiovascular development which was associated with decreased H3K36me3 [34]. Currently, in vivo functional data in the context of heart development are missing for NSD1 and ASH1L, as well as NSD3, for which no candidate variants have yet been identified.

Here, we used *Drosophila* to test the different H3K36 methyltransferases for their role in heart development. Our findings confirm the importance of ASH1L and SET2D during development for heart structure and function. Notably, *NSD* (fly homolog of mammalian *NSD1*, *NSD2*, and *NSD3*) is not expressed above detection levels in fly heart cells during early embryonic development. Further, we demonstrate the critical role of ASH1L and its cofactors, Caf1-55 and MRG15, in regulating H3K36me2 expression during fly heart development to obtain healthy heart function.

## 2. Materials and Methods

### 2.1. Drosophila Lines

All fly stocks were maintained at 25 °C on a standard diet (Meidi Laboratories, MD). *Drosophila* stocks were obtained from the Bloomington Drosophila Stock Center (Indiana University Bloomington, IN) and the Vienna Drosophila Resource Center (Vienna, Austria). The following lines were used in the experiments: UAS-*ash1*-RNAi (BDSC ID 36130 and 36803), UAS-*Set2*-RNAi (BDSC ID 42511 and 55221), UAS-*NSD*-RNAi (BDSC ID 34033 and VDRC ID 10836), UAS-*Caf1-55*-RNAi (BDSC ID 31714 and 34069), UAS-*MRG15*-RNAi (VDRC ID 110618 and 43802), UAS-*Caf1-55*-OE; *ash1*-R1288A (BDSC ID 93163), UAS-*MRG15*-OE; *ash1*-R1288A (BDSC ID 93164), UAS-*MRG15*-OE, UAS-*Caf1-55*-OE; *ash1*-R1288A (BDSC ID 93166). *w*^1118^ flies served as wild-type control. The 4X*Hand*-Gal4 driver was used to express RNAi-based silencing (-IR) and to overexpress (-OE) constructs in the heart. *Hand*-GFP and 4x*Hand*-Gal4 fly lines were generated and described previously [35].

### 2.2. Lethality at Eclosion

Eclosion lethality is evidenced by the percentage of flies expressing an RNAi-based silencing construct (straight wings) that fail to emerge as adults, relative to siblings that do not express the silencing construct (curly wings). The result is presented as a mortality index (MI) calculated as ((curly − straight)/curly) × 100. At least 400 flies were counted per genotype.

### 2.3. Single-Cell RNA Sequencing: Embryo Collection and Cell Isolation

*Drosophila melanogaster* expressing *Hand*-GFP were used to obtain embryonic heart cells, with *w*^1118^ flies as control for setting up flow cytometry. One-week-old flies were used for egg collection. To collect synchronized embryos, grape juice agar plates were changed three times, with one-hour intervals, before egg collection; eggs were collected every hour and kept at 25 °C before transfer to 18 °C for further development until the desired stages: Embryonic stages 13, 14 early (14-E), 14 late (14-L), 15, and 16.

Then, the embryos were collected and dissociated into single-cell suspension according to previously published methods [36] with some modifications. Briefly, collected embryos were washed thoroughly with ice-cold 1X phosphate-buffered saline (1XPBS) and then dechorionated in fresh 4–5% bleach (sodium hypochlorite solution; Sigma, Apotome; ZEISS, Oberkochen, Baden-Württemberg, Germany) for 3 min at room temperature. Dechorionated embryos were extensively rinsed with water. Then, embryos (~150 mg) were drained on absorbent paper and, using a paint brush, transferred to a Dounce homogenizer (15 mL Tissue Grinder; Dounce). A 5-mL suspension of embryos in Schneider medium was subjected to mechanical disruption using the Dounce homogenizer. Homogenized samples were transferred to 15-mL centrifuge tubes. Collagenase (Type I) (20 U/mL; Worthington, Columbus, OH, USA), 100 U total, was added to the Schneider medium with the homogenized embryos and the samples were incubated at room temperature for 10 min.

Next, 10 μL of ethylenediaminetetraacetic acid (EDTA) (0.5 M; final concentration of 1 mM) and 0.5 mL 2.5% trypsin were added to the 5 mL cell solution to digest the samples for another 20 min at room temperature. The trypsin reaction was stopped by adding 20% fetal bovine serum (FBS), and cells were collected using a 70 μm mesh for filtration. Next, the cells were pelleted by centrifugation (1000× *g*, 5 min, 4 °C) and resuspended in 2 mL pre-chilled artificial hemolymph. Cells were further filtered using a 40-μm mesh, resuspended in 0.5–1.0 mL, of which 10 μL was used for visual examination under a fluorescence microscope (Apotome; ZEISS, Jena, Germany). Isolated embryonic cells were sorted via flow cytometry (BD Influx cell sorter). Collected cells were pelleted via centrifugation (1000× *g*, 5 min, 4 °C) and resuspended in 50 μL pre-chilled artificial hemolymph. Cell suspensions were examined under a fluorescence microscope (Apotome; ZEISS, Germany) and cell numbers were counted with a Neubauer hemocytometer, at which point, the samples were ready for downstream experiments.

### 2.4. Single-Cell RNA Sequencing: Library Generation and Sequencing

Single-cell libraries were generated according to the manual of Chromium Single Cell 3ʹ Reagent Kits v3 (10 × Genomics). Single cells were partitioned into Gel bead-in-EMulsions (GEMs) in a Chromium Single Cell Controller (10 × Genomics) with a target cell number range of 5000–8000. Sequencing libraries were constructed according to the methods described previously [37]. Briefly, GEM samples were immediately reverse transcribed into cDNA and proceeded to downstream cleanup. The cDNAs were enzymatically fragmented and underwent end repair and 3′ A-tailing after further amplification. Adaptor ligation was performed after double-sided size selection via SPRIselect beads and followed by sample index PCR. UMI (unique molecular identifier) sequences, 10 × barcode sequences, sequencing primer P5 and P7 on both ends, and sample index sequences were all added to the cDNA samples. Finally, the library was purified and 1 μL of the amplified cDNA libraries was quality assessed and quantified via BioAnalyzer High Sensitivity Chip (Agilent, Santa Clara, CA, USA) and Qubit dsDNA HS (High Sensitivity) assay (Invitrogen, Illumina, San Diego, CA, USA). The libraries were sequenced using an Illumina HiSeq 2500 (Illumina, Tokyo, Japan).

### 2.5. Single-Cell RNA Sequencing: Data Processing and Cardiogenic Progenitors Cluster Analysis

Raw sequencing reads of Chromium single-cell 3′ RNA-seq output were processed and a data matrix was generated using the Cell Ranger pipeline version 3.0.2 (10 × Genomics, Pleasanton, CA, USA (http://10xgenomics.com) (accessed on 1 January 2020). Cell cluster analyses were performed with the Seurat package (version 3.1.2) as described in the tutorials (vignette) [38]. Previous results have reported that mitochondrial transcript abundance is much higher in tissues with high energy demands such as heart and muscle. Especially in the human heart, mitochondrial transcripts comprise almost 30% of total mRNA [39]. Therefore, we considered cells with over 30% expression of mitochondrial genes to be dead or debris. For the detection of potential doublet artifacts from our scRNA-seq result, we used DoubletFinder 2.0.3 [40] without specifying any ground-truth information. In the analysis, we used a doublet formation rate of 7.5% for the estimation of homotypic doublet proportion. We performed a global-scaling normalization method, LogNormalize, which normalizes the gene expression of each cell by the total expression, multiplies this by a scale factor (10,000 by default), and log-transforms the result in Seurat. Unsupervised uniform manifold approximation and projection (UMAP) for dimension reduction [41] was used for non-linear dimensional reduction and cluster results were visualized in two-dimensional plots. We defined the cell types based on the expression of known markers—including *tinman* (*tin*), *seven up* (*svp*), *odd skipped* (*odd*), and *even skipped* (*eve*) for cardiogenic progenitor cells, as well as checking the function and tissue distribution of those marker genes in FlyBase [42].

From this extensive dataset, we used the “Cardiogenic progenitors” cluster to determine the normalized expression (UMI counts per million mapped reads) of *absent, small, or homeotic discs 1* (*ash1*), *SET domain containing 2* (*Set2*), *Nuclear receptor binding SET domain protein* (*NSD*), *Chromatin assembly factor 1*, *p55 subunit* (*Caf1-55*), and *MORF-related gene 15* (*MRG15*).

### 2.6. Confirmation RNAi by Quantitative RT-PCR

RNA from heart tissue dissected from 60 adult flies of the relevant genotype, was isolated using TRIzol (Invitrogen) as previously described [43,44]. RNA purity and concentration were determined using a Nanodrop-1000 (Thermo Scientific, Waltham, MA, USA). Total RNA (1 µg) was transcribed into cDNA using All-In-One RT MasterMix (Applied Biological Materials, Richmond, BC, Canada). Quantitative RT-PCR was performed via real-time analysis using Power SYBR Green PCR Master Mix (Applied Biosystems, Waltham, MA, USA). The gene *Ribosomal protein L32* (*RpL32*) was used as the internal control. Sequence of the primers (Integrated DNA Technologies) used was as follows: *ash1*-Forward: TATCCACCGCCCAGGAGTAA; *ash1*-Reverse: CGTACGCTTCCGTAATCCGA; *Set2*-Forward: GCAAAAGCAAACACCGTTGC; *Set2*-Reverse: CGCTTCGATGAGGAATCCGA; *RpL32*-Forward: ACAGGCCCAAGATCGTGAAG; *RpL32*-Reverse: TGTTGTCGATACCCTTGGGC. The qRT-PCR products were quality checked via 1% agarose gel with SYBR Safe dye (Invitrogen, Waltham, MA, USA).

Quantitative values were determined using the 2-DDCT method [45] and normalized to *RpL32* as the endogenous reference gene. Values were derived from three experiments (replicates); in each, an independent pooled RNA sample (from 60 flies) was used.

### 2.7. Adult Drosophila Survival Assay

*Drosophila* larvae were kept at 25 °C to induce UAS-transgene expression. Adult male flies were subsequently maintained in vials at 25 °C, each vial containing 20 animals. Mortality was monitored every 48 h. In total, 100 flies were assayed per genotype.

### 2.8. Heart Structural Analysis: Immunochemistry

Adult *Drosophila* and 3rd instar larvae were dissected and fixed for 10 min in 4% paraformaldehyde in 1xPBS. Primary antibodies were incubated overnight at 4 °C in 2% bovine serum albumin (BSA; Sigma) with 0.1% Triton-X (Sigma) in 1xPBS (PBST). Following washes, secondary antibodies were incubated for 2 h at room temperature in PBST. Alexa Fluor R555 phalloidin was obtained from Thermo Fisher. Mouse anti-Pericardin antibody (EC11; Developmental Studies Hybridoma Bank, University of Iowa, IA) was used at 1:500 dilution, followed by Cy5-conjugated secondary antibodies (Jackson ImmunoResearch Laboratories, West Grove, PA, USA). Confocal imaging was performed using a ZEISS LSM900 microscope with a 63× Plan-Apochromat 1.4 N.A. oil objective. Images were acquired using ZEN blue edition (version 3.0). Segment A2 of the heart was imaged by collecting Z-stacks. Control groups were imaged first to establish the laser intensity and exposure time for the entire experiment. The exposure time was based on image saturation (at a set point of approximately 70% of maximum saturation) to enable the comparison of fluorescence intensity across all genotypes.

### 2.9. Heart Structural Analysis: Quantitation

*Drosophila* heart cardiac myofibril density and Pericardin deposition were quantified as previously described [46]. For quantitative comparisons, we analyzed six flies for each genotype. ImageJ software (version 1.49) [47] was used to process the images. The Z-stack projections were screened and image levels containing cardiac myofibrils were selected for analysis while avoiding the ventral muscle layer that underlies the heart tube. The cardiac myofibril number was quantified by using the MyofibrilJ plugin for Fiji (version 1.53q) [48]. The entire heart region in segment A2 was selected using the freehand selection function in Fiji to count the number of cardiac myofibrils. For quantitation, cardiac myofibril density was calculated as the cardiac myofibril number divided by the size of the heart region. Pericardin deposition was measured in heart segment A2 based on the fluorescence intensity. Cardiac myofibril density and Pericardin deposition were each normalized to the values obtained in the control flies.

### 2.10. OCT Measurements and Analysis of Cardiac Function

Cardiac function in adult *Drosophila* was measured using an optical coherence tomography (OCT) system (Bioptigen). (This system was built by the Biophotonics Group, Duke University, NC [49,50].) For this, four-day-old flies were anesthetized with carbon dioxide (CO_2_) for 3–5 min, with females preselected from each group. Each fly was gently placed on a plate with petroleum jelly (Vaseline) for immobilization with the dorsal aspect facing the OCT microscopy source, then rested for at least 10 min to ensure the fly was fully awake. For each genotype, 10 flies were tested. OCT was used to record the adult heart rhythm and heart wall movement at the same position, i.e., the cardiac chamber in abdominal segment A2, of each fly. Each measurement was obtained at three different positions within abdominal segment A2; these were averaged to obtain the cardiac diameter for that fly. M-mode images were used to record heart wall movement during the cardiac cycle. ImageJ software (version 1.49) [47] was used to process the images. The diastolic diameter and systolic diameter were processed and measured based on three consecutive heartbeats. The heart period (in seconds) was determined by counting the total number of beats during a 15-s recording, then dividing 15 by the number of beats.

### 2.11. H3K36 Methylation Marks: Corroborate Antibody Detection

Fly larval brains (*w*^1118^ control) were dissected and fixed for 10 min in 4% paraformaldehyde 1xPBS and blocked in PBST for 40 min. Rabbit anti-H3K36me1 (Abcam, Cambridge, UK; ab9048), anti-H3K36me2 (Abcam; ab9049), and anti-H3K36me3 (Abcam; ab9050) were each incubated at 1:200 dilution overnight at 4 °C, followed by incubation with Cy3-conjugated secondary antibody (Jackson ImmunoResearch Laboratories, Inc., West Grove, PA, USA) at 1:1000 dilution for 2 h at room temperature. Confocal imaging was performed using a ZEISS ApoTome.2 microscope with 63× Plan-Apochromat 1.4 N.A. oil objective. Images were acquired using ZEN blue edition (version 3.0). ImageJ software (version 1.49) was used to process the images.

### 2.12. H3K36 Methylation Marks: Immunochemistry

Adult *Drosophila* and 3rd instar larvae were dissected and fixed for 10 min in 4% paraformaldehyde in 1xPBS. Primary antibodies were incubated overnight at 4 °C in PBST. Following washes, secondary antibodies were incubated for 2 h at room temperature in PBST. Rabbit anti-H3K36me1 (Abcam; ab9048), anti-H3K36me2 (Abcam; ab9049), and anti-H3K36me3 (Abcam; ab9050) were each used at 1:200 dilution, followed by Cy3-conjugated secondary antibodies (Jackson ImmunoResearch Laboratories). Confocal imaging was performed using a ZEISS LSM900 microscope with a 63× Plan-Apochromat 1.4 N.A. oil objective. Images were acquired using ZEN blue edition (version 3.0). Segment A4 of the heart was imaged by collecting Z-stacks. Control groups were imaged first to establish the laser intensity and exposure time for the entire experiment. The exposure time was based on image saturation (at a set point of approximately 70% of maximum saturation) to enable the comparison of fluorescence intensity across all genotypes.

### 2.13. H3K36 Methylation Marks: Quantitation

For quantitative comparison of the nuclear methylation levels, cardiac cells in the 3rd instar larval heart were analyzed. A single image typically captured three cells/nuclei. We obtained images from six larvae of each genotype. For each genotype, the mean fluorescent intensity in the nucleus of ~15 heart cells total (2/3 per larvae) was used to determine the nuclear methylation level. Finally, nuclear methylation levels were normalized to the value obtained from the control flies.

### 2.14. Statistical Analysis

Statistical analysis was performed using PAST.exe software (Natural History Museum, University of Oslo (UiO), Oslo, Norway). Values are presented as mean along with the standard deviation (s.d). Student’s *t*-test was used for comparison between the two groups. Kruskal–Wallis H-test followed by a Dunn’s test was used for comparisons between multiple groups. Statistical significance was defined as *p* < 0.05.

## 3. Results

### 3.1. Silencing ash1 or Set2, Which Encode H3K36 Methyltransferases, Induced Developmental Lethality at the Pupal–Adult Stage

**Figure 1:**
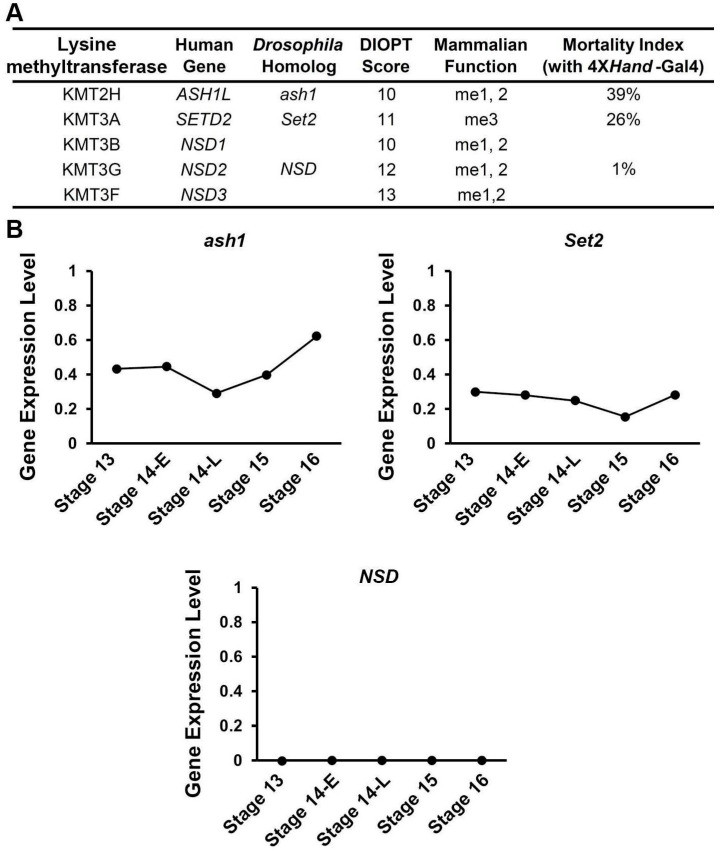
Heart-specific silencing of *ash1* or *Set2* induced pupal–adult stage developmental lethality. (**A**) Human H3K36 methyltransferases and their *Drosophila* homologs. Eclosion lethality induced by 4X*Hand*-Gal4-driven expression of UAS-RNAi transgenes targeting *absent, small, or homeotic discs 1* (*ash1*), *SET domain containing 2* (*Set2*), or *nuclear-receptor binding SET domain protein* (*NSD*). Developmental mortality attributable to RNAi heart expression (mortality rate) is calculated as ((curly − straight)/curly) × 100. Curly wings (CyO), no transgene expression; straight wings, express 4X*hand*-Gal4-driven UAS-RNAi transgene. DIOPT, DRSC Integrative Ortholog Prediction Tool (maximum score 15). Mammalian function based on existing literature [18,19,20,21,22,23,24]. (**B**) Normalized gene expression levels (UMI counts per million mapped reads) for *ash1*. *Set2*, and *NSD* in *Drosophila* heart cells (single-cell RNA sequencing) at embryonic stages 13, 14 early (14-E), 14 late (14-L), 15, and 16.

The known human H3K36 methylases have highly conserved *Drosophila* homologs (Figure 1A). Using the fly model system, we first looked at the expression levels of the H3K36 methyltransferases encoded by *ash1*, *Set2*, and *NSD* during critical stages of fly heart development, from the migration of bilateral rows of cardiac progenitors (stage 13 onward) to the formation of a closed heart tube (stage 16). Both *ash1* and *Set2* showed steady expression levels in heart cells throughout early heart development, while the expression of *NSD* did not meet detection level at any of the stages (Figure 1B). These data suggest that NSD is not involved in these earliest stages of heart development in the fly and can be used as a negative control.

**Figure 2:**
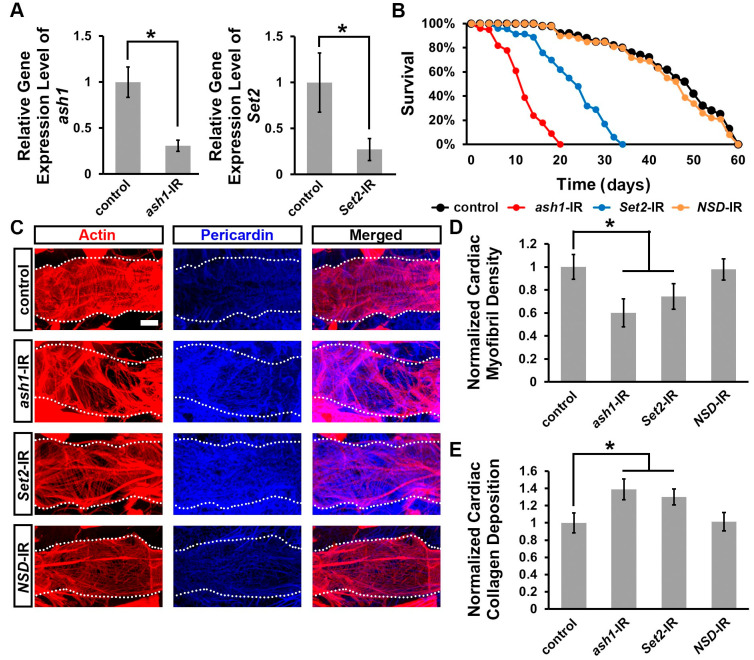
Heart-specific silencing of *ash1* or *Set2* impacted survival and cardiac defects in adult *Drosophila.* Lifespan and heart morphology for adult flies expressing *absent, small, or homeotic discs 1* (*ash1*), *SET domain containing 2* (*Set2*), or *Nuclear receptor binding SET domain protein* (*NSD*) RNAi transgenes (-IR) driven by the heart-specific 4X*hand*-Gal4. Control, wild-type (*w*^1118^) flies. (**A**) Expression of *ash1* and *Set2* in adult fly heart via real-time quantitative PCR. N = 3 replicates, 60 flies each, per genotype. Statistical significance (*) was defined as *p* < 0.05 using the Student’s *t*-test. (**B**) Survival curves. N = 100 flies per genotype. (**C**) Cardiac actin myofibers visualized via phalloidin staining (red). Pericardin detected via immunofluorescence (blue). Dotted lines delineate the outline of the heart tube. Scale bar = 20 µm. (**D**) Quantitation of adult heart cardiac myofibril density relative to control (WT, *w*^1118^). N = 6 flies per genotype. (**E**) Quantitation of adult heart cardiac collagen (Pericardin) deposition relative to control (WT, *w*^1118^). N = 6 flies per genotype. Statistical significance (*) was defined as *p* < 0.05 using Kruskal–Wallis H-test followed by a Dunn’s test.

**Figure 3:**
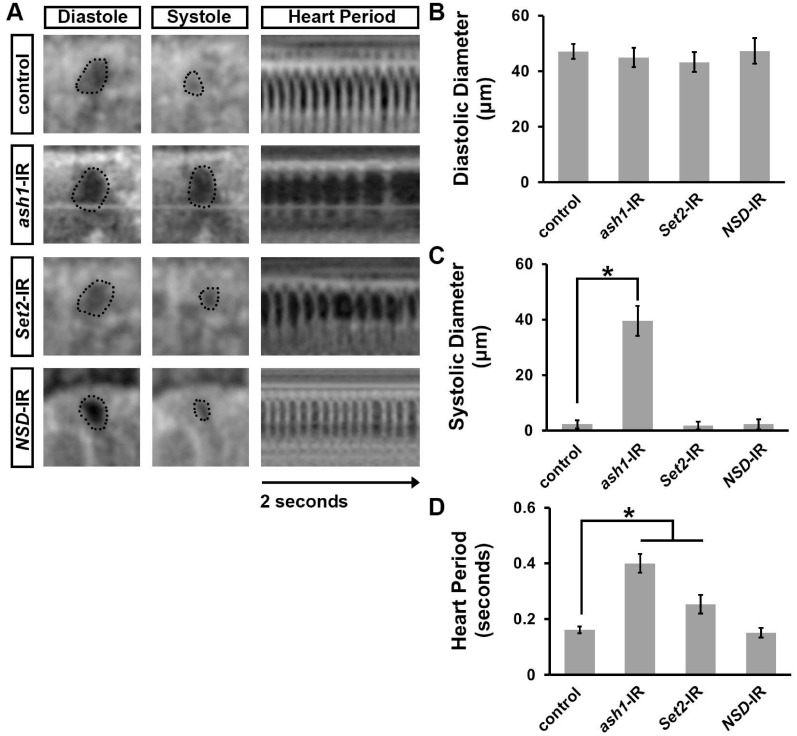
Heart-specific silencing of *ash1* or *Set2* affected cardiac function. (**A**) Images from *Drosophila* heartbeat videos obtained via optical coherence tomography (OCT). Representative images show heart function in flies that carry heart-specific (4X*hand*-Gal4) expression of UAS-RNAi transgenes (-IR) targeting *absent, small, or homeotic discs 1* (*ash1*), *SET domain containing 2* (*Set2*), or *Nuclear-receptor binding SET domain protein* (*NSD*). WT, wild-type (*w*^1118^) flies. (**B**) Quantitation of adult heart diastolic diameter. N = 10 flies per genotype. (**C**) Quantitation of adult heart systolic diameter. N = 10 flies per genotype. (**D**) Quantitation of heart period. N = 10 flies per genotype. Statistical significance (*) was defined as *p* < 0.05 using Kruskal–Wallis H-test followed by a Dunn’s test.

To study the role of genes that encode H3K36 methyltransferases during fly heart development, we employed the *Drosophila* UAS-Gal4 system with RNAi knockdown. We combined the heart-specific promoter, 4X*hand*-Gal4, with UAS-*ash1*-RNAi, UAS-*Set2*-RNAi, or UAS-*NSD*-RNAi. Quantitative real-time PCR demonstrated effective knock-down of *ash1* and *Set2* (Figure 2A). Two independent RNAi lines were tested for each gene and showed similar results; therefore, representative data for one line each have been displayed in the figures (Figure 1, Figure 2 and Figure 3, data are shown for *ash1*-IR ID 36803, *Set2*-IR ID 55221, and *NSD*-IR ID 34033). Silencing *ash1* and *Set2* caused high mortality at eclosion (39% and 26%, respectively) (Figure 1A). Consistent with the gene expression data, expressing *NSD*-RNAi in the fly heart did not affect mortality (Figure 1A). These results indicate that *ash1* and *Set2* are important for *Drosophila* heart development.

### 3.2. Silencing ash1 or Set2 Impacted Survival and Induced Cardiac Defects in Adult Drosophila

In addition to causing high mortality at eclosion (Figure 1A), silencing *ash1* or *Set2* also dramatically shortened the lifespan of those adult flies that did emerge from the *ash1*-IR and *Set2*-IR fly lines compared to wild-type (*w*^1118^) and *NSD*-IR (negative) control flies (Figure 2B).

Next, we examined the adult *Drosophila* heart for morphological defects caused by heart-specific silencing of *ash1* or *Set2*. The fly hearts were stained with phalloidin to visualize the structure of the cardiac actin filaments. As expected, expressing *NSD*-IR in the fly heart did not lead to any cardiac morphological defects (Figure 2C–E). Silencing *ash1* or *Set2* was associated with overall disorganization of the cardiac actin filaments (Figure 2C) and reduced cardiac myofibril density (Figure 2D). We also observed increased deposition of Pericardin (Figure 2C,E), a type IV collagen that plays a critical role in maintaining *Drosophila* cardiac tissue integrity. The overabundance of Pericardin indicates the pathophysiological condition fibrosis [51,52,53,54].

### 3.3. Silencing ash1 or Set2 Caused Cardiac Functional Defects in Adult Drosophila

To assess cardiac functional defects induced by silencing *ash1* or *Set2*, we applied optical coherence tomography (OCT). The orthogonal view of the heart provides accurate and real-time measurements of the heart tube diameter and heart period (Figure 3A). Silencing *ash1*, but not *Set2*, was associated with an increased systolic diameter; neither showed a change in diastolic diameter (Figure 3A–C). Compared to wild-type (*w*^1118^) control flies, the heart period in *ash1-* and *Set2*-gene-silenced flies was significantly increased (Figure 3D). As expected, heart function in *NSD*-IR (negative control) flies was indistinguishable from that in wild-type (*w*^1118^) control flies (Figure 3A–D). Thus, in addition to heart structural integrity, the H3K36 methyltransferases encoded by *ash1* and *Set2* are required for normal heart function.

### 3.4. Silencing Caf1-55 or MRG15, Which Encode Ash1-Complex Components, Increased Developmental Lethality at the Pupal–Adult Stage, Reduced Lifespan, and Caused Heart Morphological Defects

**Figure 4:**
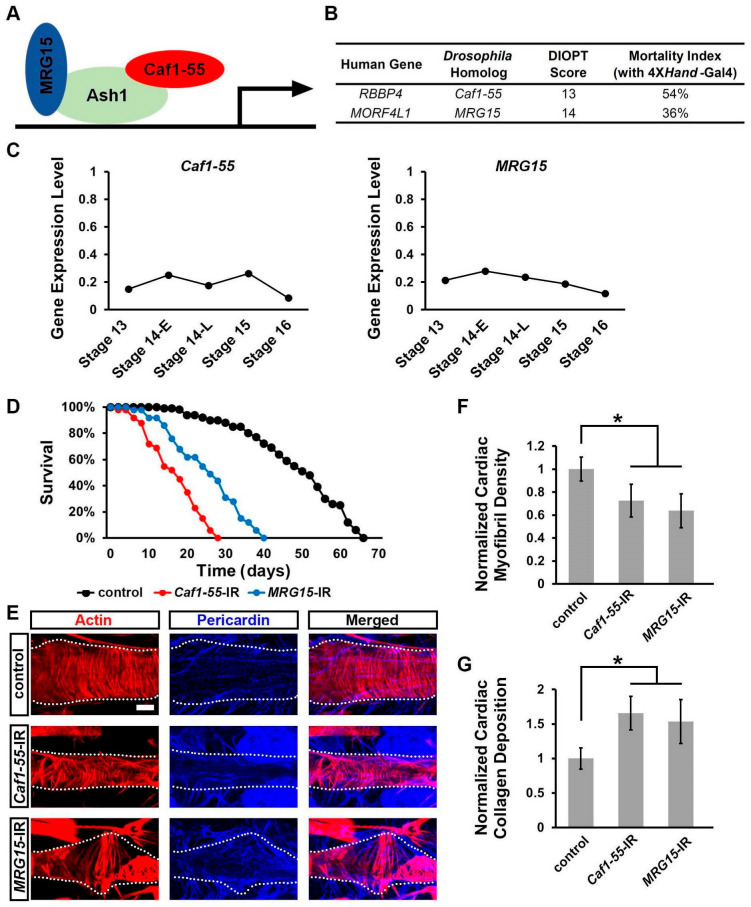
Heart-specific silencing of *Caf1-55* or *MRG15* led to pupal–adult stage developmental lethality, impacted survival, and induced cardiac defects in adult *Drosophila.* Lifespan and heart morphology for adult flies expressing *Chromatin assembly factor 1*, *p55 subunit* (*Caf1-55*) or *MORF-related gene 15* (*MRG15*) RNAi transgenes (-IR) driven by the heart-specific 4X*hand*-Gal4. WT, wild-type (*w*^1118^) flies. (**A**) Graphic of the Ash1 protein complex with cofactors, Caf1-55 and MRG15. (**B**) Human ASH1L cofactors and their *Drosophila* homologs. Eclosion lethality induced by 4X*hand*-Gal4-driven expression of UAS-RNAi transgenes targeting *Caf1-55* or *MRG15*. Developmental mortality attributable to RNAi heart expression (Mortality rate) is calculated as (curly − straight)/curly) × 100. Curly wings (CyO)—no transgene expression; straight wings express 4X*Hand*-Gal4-driven UAS-RNAi transgene. DIOPT, DRSC Integrative Ortholog Prediction Tool (maximum score 15). (**C**) Normalized gene expression levels (UMI counts per million mapped reads) for *Caf1-55* and *MRG15* in *Drosophila* heart cells (single-cell RNA sequencing) at embryonic stages 13, 14 early (14-E), 14 late (14-L), 15, and 16. (**D**) Survival curves. N = 100 flies per genotype. (**E**) Cardiac actin myofibers visualized via phalloidin staining (red). Pericardin detected via immunofluorescence (blue). Dotted lines delineate the outline of the heart tube. Scale bar = 20 µm. (**F**) Quantitation of adult heart cardiac myofibril density relative to control (WT, *w*^1118^). N = 6 flies per genotype. (**G**) Quantitation of adult heart cardiac collagen (Pericardin) deposition relative to control (WT, *w*^1118^). N = 6 flies per genotype. Statistical significance (*) was defined as *p* < 0.05 using Kruskal–Wallis H-test followed by a Dunn’s test.

**Figure 5:**
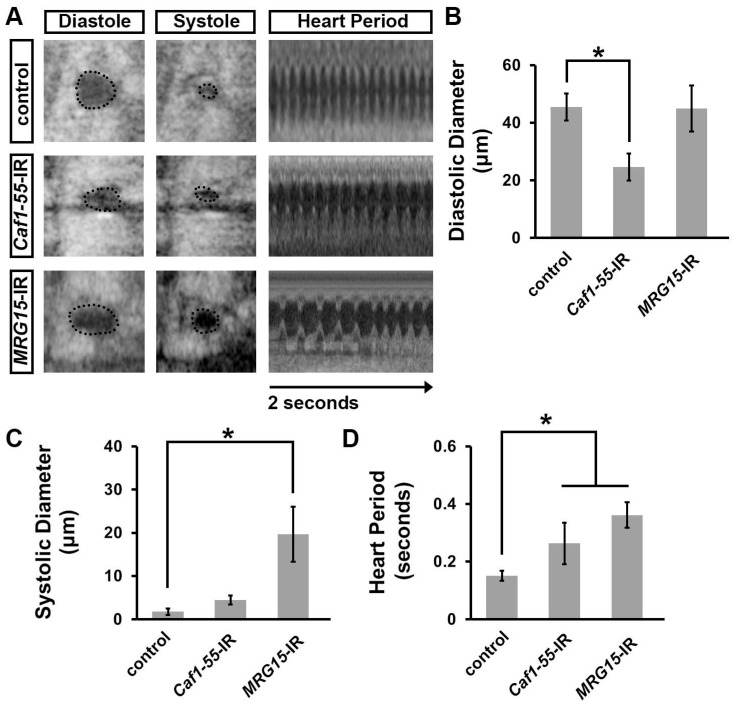
Heart-specific silencing of *Caf1-55* and *MRG15* affected cardiac function. (**A**) Images from *Drosophila* heartbeat videos obtained via optical coherence tomography (OCT). Representative images show heart function in flies that carry heart-specific (4X*Hand*-Gal4) expression of UAS-RNAi transgenes (-IR) targeting *Chromatin assembly factor 1*, *p55 subunit* (*Caf1-55*) or *MORF-related gene 15* (*MRG15*). WT, wild-type (*w*^1118^) flies. (**B**) Quantitation of adult heart diastolic diameter. N = 10 flies per genotype. (**C**) Quantitation of adult heart systolic diameter. N = 10 flies per genotype. (**D**) Quantitation of heart period. N = 10 flies per genotype. Statistical significance (*) was defined as *p* < 0.05 using Kruskal–Wallis H-test followed by a Dunn’s test.

Previous studies have shown that Ash1 depends on complex formation with Caf1-55 (also known as NURF55) and MRG15 to induce H3K36 methylation activity [55] (Figure 4A). Therefore, we next investigated whether these two cofactors are also required during *Drosophila* heart development. Both genes were expressed throughout early heart development (Figure 4C). We combined the heart-specific promoter, 4X*hand*-Gal4, with UAS-*Caf1-55*-RNAi or UAS-*MRG15*-RNAi to obtain heart-specific knockdown. Two independent RNAi lines were tested for each gene and showed similar results; therefore, representative data for one line each have been displayed in the figures (Figure 4 and Figure 5; data shown for *Caf1-55*-IR ID 34069 and *MRG15*-IR ID 110618). Silencing *Caf1-55* or *MRG15* caused high mortality at eclosion (54% and 36%, respectively) (Figure 4B). We also observed dramatically shortened lifespans for those adult flies that did emerge from the *Caf1-55*-IR and *MRG15*-IR fly lines compared to wild-type (*w*^1118^) control flies (Figure 4D).

Next, we examined the adult *Drosophila* heart for morphological defects caused by heart-specific silencing of *Caf1-55* or *MRG15*. Silencing either Ash1 cofactor caused disorganized cardiac actin filaments (Figure 4E) and reduced cardiac myofibril density (Figure 4F). We also observed increased deposition of collagen (Pericardin) (Figure 4E,G), indicative of fibrosis [51,52,53,54]. These data show that both Ash1-complex components, Caf1-55 and MRG15, are required for heart development, as deficiency for either leads to severe cardiac structural defects.

### 3.5. Silencing Caf1-55 or MRG15 Caused Cardiac Functional Defects in Adult Drosophila

To assess cardiac functional defects induced by silencing *Caf1-55* or *MRG15*, we applied OCT. Silencing *Caf1-55*, but not *MRG15*, was associated with reduced diastolic diameter (Figure 5A,B); whereas silencing *MRG15*, but not *Caf1-55*, increased systolic diameter (Figure 5A,C). Meanwhile, silencing either Ash1 cofactor significantly increased the heart period compared to wild-type (*w*^1118^) control flies (Figure 5A,D). These data demonstrate that, in addition to heart structural integrity, both Ash1-complex components—Caf1-55 and MRG15—are necessary for normal heart function.

### 3.6. Simultaneous Overexpression of Caf1-55 and MRG15 Rescued Cardiac Functional Defects Caused by ash1 Mutation

**Figure 6:**
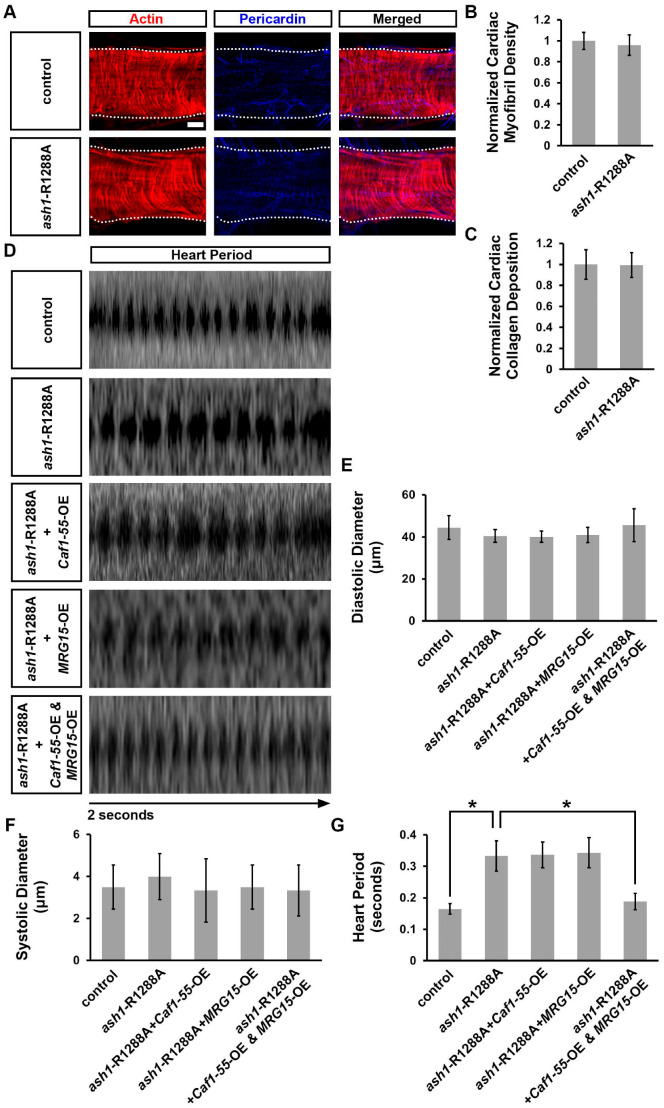
Simultaneous, heart-specific overexpression of *Caf1-55* and *MRG15* rescued the cardiac defect caused by the *ash1*-R1288A mutant. (**A**) Adult heart phenotype induced by the *absent, small, or homeotic discs 1* (*ash1*)-R1288A homozygous mutant. WT, wild-type (*w*^1118^) flies. Cardiac actin myofibers visualized via phalloidin staining (red). Pericardin detected via immunofluorescence (blue). Dotted lines delineate the outline of the heart tube. Scale bar = 20 µm. (**B**) Quantitation of adult heart cardiac myofibril density relative to control (WT, *w*^1118^). N = 6 flies per genotype. (**C**) Quantitation of adult heart cardiac collagen (Pericardin) deposition relative to control (WT, *w*^1118^). N = 6 flies per genotype. (**D**) Images from *Drosophila* heartbeat videos obtained via optical coherence tomography (OCT). Representative images show heart function in flies that carry heart-specific (4X*Hand*-Gal4) expression of the *ash1*-R1288A homozygous mutant, with or without heart-specific (4X*Hand*-Gal4) UAS-overexpression (-OE) of *Chromatin assembly factor 1*, *p55 subunit* (*Caf1-55*) or *MORF-related gene 15* (*MRG15*) transgenes. (**E**) Quantitation of adult heart diastolic diameter. N = 10 flies per genotype. (**F**) Quantitation of adult heart systolic diameter. N = 10 flies per genotype. (**G**) Quantitation of heart period. N = 10 flies per genotype. Statistical significance (*) was defined as *p* < 0.05 using Student’s *t*-test for two groups or Kruskal–Wallis H-test followed by a Dunn’s test for multiple groups.

Previous study has shown that MRG15 binds to Ash1 at R1288 to stimulate H3K36 methyltransferase activity [55]. Therefore, to further understand the genetic interaction among Ash1, Caf1-55, and MRG15 during *Drosophila* heart development, we used the published Cas9/CRISPR-generated knock-in *ash1*-R1288A fly line, which disrupts the Ash1−MRG15 binding site [55]. In our assays, this *ash1*-R1288A homozygous mutant fly displayed no cardiac structural defects including organization of the cardiac actin filaments, cardiac myofibril density, and Pericardin deposition (Figure 6A–C). Nor did these flies show changes to their heart diameter during diastole (relaxation) or systole (contraction) (Figure 6D–F). However, we did observe a significantly increased heart period in these flies, a cardiac functional defect (Figure 6D,G). The much milder phenotype of the *ash1*-R1288A knock-in flies compared to the *ash1*-IR flies (Figure 1, Figure 2 and Figure 3) is likely due to residual functionality, rather than the null with knockdown, as the mutation only weakens Ash1 binding to MRG15 [55]. Using the heart-specific promoter 4X*Hand*-Gal4 to overexpress either *Caf1-55* or *MRG15* in *ash1*-R1288A homozygous mutant flies did not rescue the increased heart period (Figure 6D,G). However, overexpressing *Caf1-55* and *MRG15* simultaneously did rescue this cardiac functional defect (Figure 6D,G). These findings, combined with the previously published co-IP data [55], support direct interaction between Ash1 and MRG15 and show that both complex components, MRG15 and Caf1-55, are important for Ash1‘s role in heart function.

### 3.7. Simultaneous Overexpression of Caf1-55 and MRG15 Restored the Reduced H3K36me2 Marks Caused by ash1 Mutation

**Figure 7:**
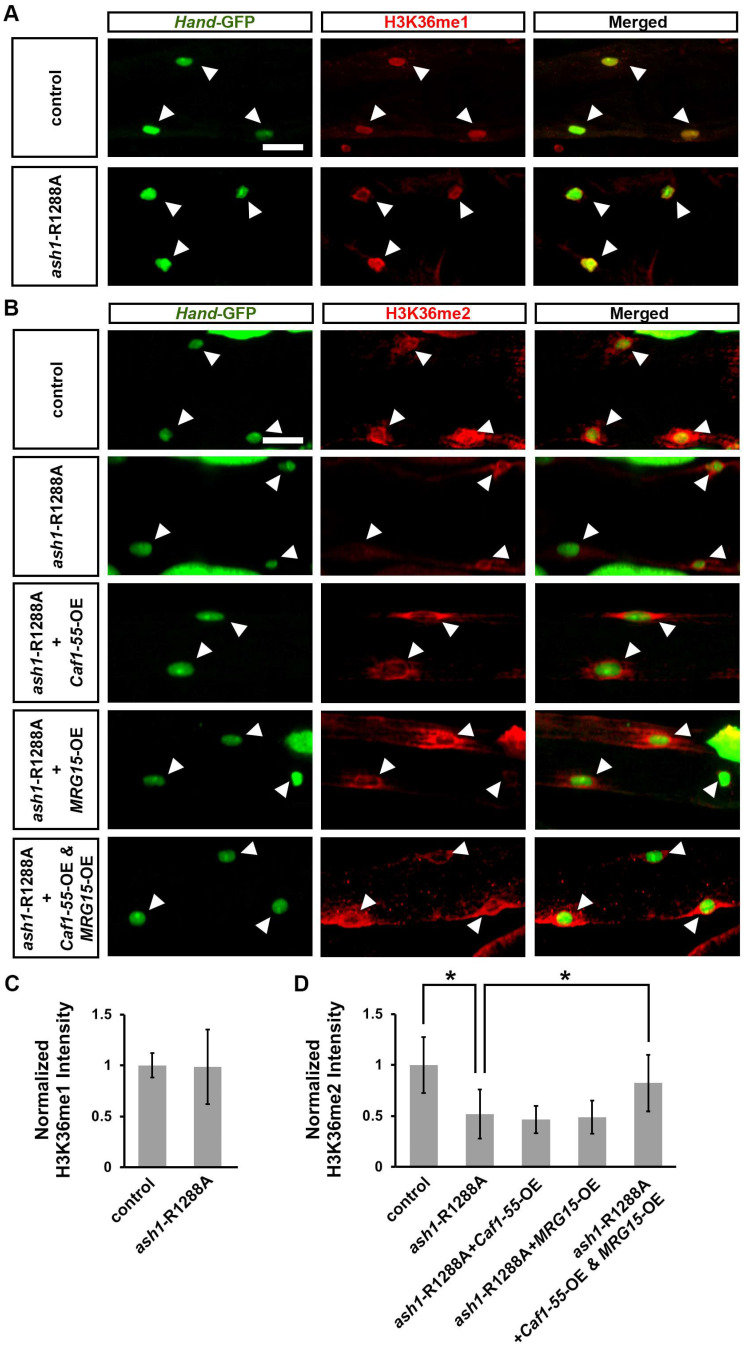
Simultaneous, heart-specific overexpression of *Caf1-55* and *MRG15* restored H3K36 dimethylation level reduction caused by the *ash1*-R1288A mutant. (**A**) Larval heart H3K36 monomethylation (H3K36me1; red) detected via immunofluorescence in *absent, small, or homeotic discs 1* (*ash1*)-R1288A homozygous mutant and wild-type (WT, *w*^1118^;*Hand*-GFP) flies. Arrowhead points to the nucleus of a heart cell (green; *Hand*-GFP). Scale bar = 20 µm. (**B**) Larval heart H3K36 dimethylation (H3K36me2; red) detected by immunofluorescence in *ash1*-R1288A homozygous mutant flies with or without heart-specific (4X*Hand*-Gal4) UAS-overexpression transgenes (-OE) targeting *Chromatin assembly factor 1*, *p55 subunit* (*Caf1-55*) or *MORF-related gene 15* (*MRG15*), or both simultaneously, as well as wild-type (WT, *w*^1118^;*Hand*-GFP) flies. Arrowhead points to the nucleus of a heart cell (green; *Hand*-GFP). Scale bar = 20 µm. (**C**) Quantitation of larval heart cardiac H3K36me1 marks relative to that in control (WT, *w*^1118^) flies. N = 15 nuclei from 6 larvae per genotype. (**D**) Quantitation of larval heart cardiac H3K36me2 marks relative to that in control (WT, *w*^1118^) flies. N = 15 nuclei from 6 larvae per genotype. Statistical significance (*) was defined as *p* < 0.05 using Student’s *t*-test for two groups or Kruskal–Wallis H-test followed by a Dunn’s test for multiple groups.

To understand the mechanism behind the rescue of the *ash1*-R1288A-induced heart period defect by simultaneous overexpression of *Caf1-55* and *MRG15*, we investigated H3K36 methylation marks in the *Drosophila* developmental heart (images to corroborate antibody detection are in Supplemental Figure S1). By itself, the *ash1*-R1288A homozygous mutant significantly reduced H3K36 dimethylation (H3K36me2), but not H3K36 monomethylation (H3K36me1), marks in larval cardiac cells (Figure 7A–D). We did not detect H3K36 trimethylation (H3K36me3) marks in the larval cardiac cells (*data not shown*). Notably, overexpression of *Caf1-55* and *MRG15* together, but not *Caf1-55* or *MRG15* alone, restored the H3K36me2 marks in the *ash1*-R1288A homozygous mutant fly larvae to within normal range (Figure 7B,D). These data show that Caf1-55 and MRG15 combined are required for Ash1 H3K36me2 methyltransferase activity in the heart.

## 4. Discussion

We set out to validate the roles of specific H3K36 methyltransferases during heart development. Our data show that both Set2 (SETD2 in mammals) and Ash1 (ASH1L in mammals) are required during early heart development, as cardiac deficiency for either resulted in early lethality and a reduced lifespan in *Drosophila*. The hearts of these flies displayed structural and functional abnormalities. Notably, we could not detect expression of *NSD* in fly heart cells during early embryonic stages of cardiac development. These findings are in line with previous reports that have associated variants in *NSD1* and *NSD2* with congenital heart disease [12,29,30], albeit with largely circumstantial evidence that linked variants to complex neurodevelopmental disorders the subpopulations of which displayed cardiac defects [25,26,27,28,31]. *SETD2* is expressed in human fetal heart tissue [8] and is highly expressed in the mouse heart at embryonic stages (*Setd2*), where it plays a leading role in H3K36me3 methylation [34]. Like the flies, mice deficient in *Setd2* in cardiac progenitors showed early lethality (fetal, mid-gestation) and showed severe cardiovascular defects; these were accompanied by reduced H3K36me3 and reduced expression of key cardiac-related genes [34]. Variants in *ASH1L* have been associated with congenital heart disease in patients [11,12,30]. *ASH1L* is expressed in both fetal and adult human heart tissue [8], and was differentially expressed in cardiac tissue from patients with dilated cardiomyopathy (with heart failure) [6]. The data presented here support a role for ASH1L in heart development and function and establish *Drosophila* Ash1 as the functional homolog of human ASH1L in the heart.

Two Ash1 complex components, Caf1-55 (also known as NURF55) and MRG15, were first identified using *Drosophila*; this complex is conserved in mammals [55]. MRG15 stimulates the enzymatic activity of Ash1 (in vitro) and is recruited by Ash1 to target sites on the genome. Moreover, MRG15 facilitates the association of Ash1 with chromatin and the deposition of H3K36me2. Disrupting this Ash1−MRG15 interaction in flies led to gross morphological transformation of the legs and wings. This phenotype could be partially restored by overexpressing a Caf1-55−MRG15-fusion protein [55]. Likewise, in our study, the functional defects caused by *ash1*-deficiency could be rescued by overexpressing both its cofactors, *Caf1-55* and *MRG15*, simultaneously, whereas knockdown of either gene encoding an Ash1-complex component, *Caf1-55* or *MRG15*, led to heart functional defects. On the other hand, the *ash1*-R1288A point mutation weakens the binding of Ash1 to MRG15 [55], thus leaving residual complex functionality which resulted in a milder cardiac phenotype. Like the study in fly S2 cells, we found that introducing *ash1*-R1288A to the fly heart disrupted H3K36me2 methylation (significantly reduced) in fly larval cardiac cells, but we detected no change in H3K36me1 marks. Of note, the H3K36me3 marks in the fly heart were below the detection limit and might not be relevant during this stage of cardiac development.

Our data show that Caf1-55 and MRG15 combined are required for Ash1 H3K36me2 methyltransferase activity in the heart, which is important for heart function. *Drosophila* Ash1 is a trithorax-group (trxG) regulator that antagonizes polycomb repression at homeobox genes (*Hox*) mediated by H3K36me2 deposition [56]. The Hox transcription factors provide a highly conserved system for embryonic patterning, i.e., head–tail axis [56], and are key in coordinating cardiac progenitor cell patterning and differentiation [57]. Therefore, further study to determine the role of Ash1−Caf1-55−MRG15-mediated H3K36 methylation activity on Hox genes during heart development and disease is warranted. The current study has been limited by focusing on individual H3K36 methyl transferases. A previous report suggested that Caf1-55 acts on top of the HeK36me2/me3 landscape generated by NSD and Set2 [56]. It is currently unclear how interaction between the different H3K36 methylases and demethylases coordinates heart development. Finally, in addition to methylation activity at H3K36, ASH1L also displays H3K4 activity [58]. Possible interactions between different methylation marks during heart development, especially considering their purported dual regulatory role (i.e., the ability to activate and repress transcription) [8], could shed further light on the complex role of ASH1L during heart development and disease. Ultimately, to obtain the full picture we need to study the entire methylation (epigenomic) landscape, including the writers, readers, erasers, and the temporal dynamics of the methylation marks.

## Data Availability

Data and materials that support the findings of this study are available from the corresponding authors upon reasonable request. The scRNA-seq source data, including sequence reads and single-cell expression matrices have been deposited in NCBI’s Gene Expression Omnibus and are accessible through GEO accession number: GSE168774.

## Supplementary Materials

The following supporting information can be downloaded at: https://www.mdpi.com/article/10.3390/jcdd10070307/s1, Supplemental Figure S1. Expression level of H3K36me1, me2, and me3 in *Drosophila* larval ventral nerve cord. H3K36 mono- (H3K36me1), di- (H3K36me2) and trimethylation (H3K36me3) detected via immunofluorescence (red) in larval ventral nerve cord of control (*w*^1118^) flies. Boxed area is magnified in image below. Scale bar = 30 μm; Scale bar (magnification) = 4 μm.

## References

[1] Baujat G., Cormier-Daire V. (2007). Sotos Syndrome. Orphanet J. Rare Dis..

[2] Bergemann A.D., Cole F., Hirschhorn K. (2005). The Etiology of Wolf-Hirschhorn Syndrome. Trends Genet..

[3] Butler A., Hoffman P., Smibert P., Papalexi E., Satija R. (2018). Integrating Single-Cell Transcriptomic Data across Different Conditions, Technologies, and Species. Nat. Biotechnol..

[4] Cecconi M., Forzano F., Milani D., Cavani S., Baldo C., Selicorni A., Pantaleoni C., Silengo M., Ferrero G., Scarano G. (2005). Mutation analysis of theNSD1 gene in a group of 59 patients with congenital overgrowth. Am. J. Med. Genet. Part A.

[5] Chen F., Chen J., Wang H., Tang H., Huang L., Wang S., Wang X., Fang X., Liu J., Li L. (2021). Histone Lysine Methyltransferase SETD2 Regulates Coronary Vascular Development in Embryonic Mouse Hearts. Front. Cell Dev. Biol..

[6] Choma M.A., Izatt S.D., Wessells R.J., Bodmer R., Izatt J.A. (2006). Images in Cardiovascular Medicine: In Vivo Imaging of the Adult Drosophila Melanogaster Heart with Real-Time Optical Coherence Tomography. Circulation.

[7] Davis K., Azarcon P., Hickenlooper S., Bia R., Horiuchi E., Szulik M.W., Franklin S. (2021). The Role of Demethylases in Cardiac Development and Disease. J. Mol. Cell. Cardiol..

[8] Dillon S.C., Zhang X., Trievel R.C., Cheng X. (2005). The SET-domain protein superfamily: Protein lysine methyltransferases. Genome Biol..

[9] Edmunds J.W., Mahadevan L.C., Clayton A.L. (2008). Dynamic histone H3 methylation during gene induction: HYPB/Setd2 mediates all H3K36 trimethylation. EMBO J..

[10] Fu Y., Huang X., Zhang P., van de Leemput J., Han Z. (2020). Single-cell RNA sequencing identifies novel cell types in Drosophila blood. J. Genet. Genom..

[11] Gregory G.D., Vakoc C.R., Rozovskaia T., Zheng X., Patel S., Nakamura T., Canaani E., Blobel G.A. (2007). Mammalian ASH1L Is a Histone Methyltransferase That Occupies the Transcribed Region of Active Genes. Mol. Cell. Biol..

[12] Gupta H.P., Fatima M.-U., Pandey R., Ram K.R. (2023). Adult exposure of atrazine alone or in combination with carbohydrate diet hastens the onset/progression of type 2 diabetes in Drosophila. Life Sci..

[13] Han Z., Olson E.N. (2005). Hand Is a Direct Target of Tinman and GATA Factors during Drosophila Cardiogenesis and Hematopoiesis. Development.

[14] Homsy J., Zaidi S., Shen Y., Ware J.S., Samocha K.E., Karczewski K.J., DePalma S.R., McKean D., Wakimoto H., Gorham J. (2015). De novo mutations in congenital heart disease with neurodevelopmental and other congenital anomalies. Science.

[15] Huang C., Yang F., Zhang Z., Zhang J., Cai G., Li L., Zheng Y., Chen S., Xi R., Zhu B. (2017). Mrg15 stimulates Ash1 H3K36 methyltransferase activity and facilitates Ash1 Trithorax group protein function in Drosophila. Nat. Commun..

[16] Huang X., Liu Y., Wang Y., Bailey C., Zheng P., Liu Y. (2021). Dual Targeting Oncoproteins MYC and HIF1α Regresses Tumor Growth of Lung Cancer and Lymphoma. Cancers.

[17] Hughes C., Turner S., Andrews R., Vitkin A., Jacobs J. (2020). Matrix metalloproteinases regulate ECM accumulation but not larval heart growth in Drosophila melanogaster. J. Mol. Cell. Cardiol..

[18] Husmann D., Gozani O. (2019). Histone lysine methyltransferases in biology and disease. Nat. Struct. Mol. Biol..

[19] Ji W., Ferdman D., Copel J., Scheinost D., Shabanova V., Brueckner M., Khokha M.K., Ment L.R. (2020). De novo damaging variants associated with congenital heart diseases contribute to the connectome. Sci. Rep..

[20] Jin S.C., Homsy J., Zaidi S., Lu Q., Morton S., DePalma S.R., Zeng X., Qi H., Chang W., Sierant M.C. (2017). Contribution of Rare Inherited and de Novo Variants in 2,871 Congenital Heart Disease Probands. Nat. Genet..

[21] Jones R.S., Gelbart W.M. (1993). The Drosophila Polycomb-Group Gene Enhancer of zeste Contains a Region with Sequence Similarity to trithorax. Mol. Cell. Biol..

[22] Larkin A., Marygold S.J., Antonazzo G., Attrill H., dos Santos G., Garapati P.V., Goodman J.L., Gramates L.S., Millburn G., Strelets V.B. (2021). FlyBase: Updates to the *Drosophila melanogaster* knowledge base. Nucleic Acids Res..

[23] Li X., Fan H., Song X., Song B., Liu W., Dong R., Zhang H., Guo S., Liang H., Schrodi S.J. (2023). DNA methylome and transcriptome profiling reveal key electrophysiology and immune dysregulation in hypertrophic cardiomyopathy. Epigenetics.

[24] Li Y., Trojer P., Xu C.F., Cheung P., Kuo A., Drury W.J., Qiao Q., Neubert T.A., Xu R.M., Gozani O. (2009). The Target of the NSD Family of Histone Lysine Methyltransferases Depends on the Nature of the Substrate. J. Biol. Chem..

[25] Liu Y., Morley M., Brandimarto J., Hannenhalli S., Hu Y., Ashley E.A., Tang W.W., Moravec C.S., Margulies K.B., Cappola T.P. (2015). RNA-Seq identifies novel myocardial gene expression signatures of heart failure. Genomics.

[26] Livak K.J., Schmittgen T.D. (2001). Analysis of Relative Gene Expression Data Using Real-Time Quantitative PCR and the 2(-Delta Delta C(T)) Method. Methods.

[27] Lucio-Eterovic A.K., Singh M.M., Gardner J.E., Veerappan C.S., Rice J.C., Carpenter P.B. (2010). Role for the nuclear receptor-binding SET domain protein 1 (NSD1) methyltransferase in coordinating lysine 36 methylation at histone 3 with RNA polymerase II function. Proc. Natl. Acad. Sci. USA.

[28] McGinnis C.S., Murrow L.M., Gartner Z.J. (2019). DoubletFinder: Doublet Detection in Single-Cell RNA Sequencing Data Using Artificial Nearest Neighbors. Cell Syst..

[29] McInnes L., Healy J., Saul N., Großberger L. (2018). UMAP: Uniform Manifold Approximation and Projection. J. Open Source Softw..

[30] Mercer T.R., Neph S., Dinger M.E., Crawford J., Smith M.A., Shearwood A.-M.J., Haugen E., Bracken C.P., Rackham O., Stamatoyannopoulos J.A. (2011). The Human Mitochondrial Transcriptome. Cell.

[31] Petrossian T.C., Clarke S.G. (2011). Uncovering the Human Methyltransferasome. Mol. Cell. Proteom..

[32] Priest J.R., Osoegawa K., Mohammed N., Nanda V., Kundu R., Schultz K., Lammer E.J., Girirajan S., Scheetz T., Waggott D. (2016). De Novo and Rare Variants at Multiple Loci Support the Oligogenic Origins of Atrioventricular Septal Heart Defects. PLoS Genet..

[33] Ren J., Zeng Q., Wu H., Liu X., Guida M.C., Huang W., Zhai Y., Li J., Ocorr K., Bodmer R. (2023). Deacetylase-Dependent and -Independent Role of HDAC3 in Cardiomyopathy. J. Cell. Physiol..

[34] Salmand P.-A., Iché-Torres M., Perrin L. (2011). Tissue-specific cell sorting from Drosophila embryos: Application to gene expression analysis. Fly.

[35] Schmähling S., Meiler A., Lee Y., Mohammed A., Finkl K., Tauscher K., Israel L., Borath M., Philippou-Massier J., Blum H. (2018). Regulation and function of H3K36 di-methylation by the trithorax-group protein complex AMC. Development.

[36] Schneider C.A., Rasband W.S., Eliceiri K.W. (2012). NIH Image to ImageJ: 25 Years of image analysis. Nat. Methods.

[37] Spletter M.L., Barz C., Yeroslaviz A., Zhang X., Lemke S.B., Bonnard A., Brunner E., Cardone G., Basler K., Habermann B.H. (2018). A transcriptomics resource reveals a transcriptional transition during ordered sarcomere morphogenesis in flight muscle. Elife.

[38] Stassen M.J., Bailey D., Nelson S., Chinwalla V., Harte P.J. (1995). The Drosophila trithorax proteins contain a novel variant of the nuclear receptor type DNA binding domain and an ancient conserved motif found in other chromosomal proteins. Mech. Dev..

[39] Stefanovic S., Laforest B., Desvignes J.-P., Lescroart F., Argiro L., Maurel-Zaffran C., Salgado D., Plaindoux E., De Bono C., Pazur K. (2020). Hox-dependent coordination of mouse cardiac progenitor cell patterning and differentiation. Elife.

[40] Sweet M.E., Cocciolo A., Slavov D., Jones K.L., Sweet J.R., Graw S.L., Reece T.B., Ambardekar A.V., Bristow M.R., Mestroni L. (2018). Transcriptome analysis of human heart failure reveals dysregulated cell adhesion in dilated cardiomyopathy and activated immune pathways in ischemic heart failure. BMC Genom..

[41] Szulik M.W., Davis K., Bakhtina A., Azarcon P., Bia R., Horiuchi E., Franklin S. (2020). Transcriptional regulation by methyltransferases and their role in the heart: Highlighting novel emerging functionality. Am. J. Physiol. Circ. Physiol..

[42] Tanaka Y., Katagiri Z.-I., Kawahashi K., Kioussis D., Kitajima S. (2007). Trithorax-group protein ASH1 methylates histone H3 lysine 36. Gene.

[43] Taylor-Papadimitriou J., Burchell J.M. (2022). Histone Methylases and Demethylases Regulating Antagonistic Methyl Marks: Changes Occurring in Cancer. Cells.

[44] Tschiersch B., Hofmann A., Krauss V., Dorn R., Korge G., Reuter G. (1994). The protein encoded by the Drosophila position-effect variegation suppressor gene Su(var)3-9 combines domains of antagonistic regulators of homeotic gene complexes. EMBO J..

[45] TsNoreau D.R., Al-Ata J., Jutras L., Teebi A.S. (1999). Congenital Heart Defects in Sotos Syndrome. Am. J. Med. Genet..

[46] Vaughan L., Marley R., Miellet S., Hartley P.S. (2018). The impact of SPARC on age-related cardiac dysfunction and fibrosis in Drosophila. Exp. Gerontol..

[47] Wagner E.J., Carpenter P.B. (2012). Understanding the language of Lys36 methylation at histone H3. Nat. Rev. Mol. Cell Biol..

[48] Xiong Q., Zhou Y., Zhang S., Zhang Y., Xu Y., Yang Y., Zhou C., Zeng Z., Han J., Zhu Q. (2023). NSD3, a member of nuclear receptor-binding SET domain family, is a potential prognostic biomarker for pancreatic cancer. Cancer Med..

[49] Yang K.C., Yamada K.A., Patel A.Y., Topkara V.K., George I., Cheema F.H., Ewald G.A., Mann D.L., Nerbonne J.M. (2014). Deep RNA Sequencing Reveals Dynamic Regulation of Myocardial Noncoding RNAs in Failing Human Heart and Remodeling with Mechanical Circulatory Support. Circulation.

[50] Yelbuz T.M., Choma M.A., Thrane L., Kirby M.L., Izatt J.A. (2002). Optical Coherence Tomography: A New High-Resolution Imaging Technology to Study Cardiac Development in Chick Embryos. Circulation.

[51] Zaghi M., Broccoli V., Sessa A. (2019). H3K36 Methylation in Neural Development and Associated Diseases. Front. Genet..

[52] Zaidi S., Choi M., Wakimoto H., Ma L., Jiang J., Overton J.D., Romano-Adesman A., Bjornson R.D., Breitbart R.E., Brown K.K. (2013). De novo mutations in histone-modifying genes in congenital heart disease. Nature.

[53] Zanoni P., Steindl K., Sengupta D., Joset P., Bahr A., Sticht H., Lang-Muritano M., Al E., Zweier M., Ganzi O. (2021). Loss-of-function and missense variants in NSD2 cause decreased methylation activity and are associated with a distinct developmental phenotype. Genet. Med. Off. J. Am. Coll. Med. Genet..

[54] Di Zhang D., Deng Y., Kukanja P., Agirre E., Bartosovic M., Dong M., Ma C., Ma S., Su G., Bao S. (2023). Spatial epigenome–transcriptome co-profiling of mammalian tissues. Nature.

[55] Zhao T., Huang X., Han L., Wang X., Cheng H., Zhao Y., Chen Q., Chen J., Cheng H., Xiao R. (2012). Central Role of Mitofusin 2 in Autophagosome-Lysosome Fusion in Cardiomyocytes. J. Biol. Chem..

[56] Zhou X.-L., Zhu R.-R., Wu X., Xu H., Li Y.-Y., Xu Q.-R., Liu S., Huang H., Xu X., Wan L. (2019). NSD2 promotes ventricular remodelling mediated by the regulation of H3K36me2. J. Cell. Mol. Med..

[57] Zhu J.-Y., Fu Y., Nettleton M., Richman A., Han Z. (2017). High throughput in vivo functional validation of candidate congenital heart disease genes in Drosophila. Elife.

[58] Zhu J.-Y., Fu Y., Richman A., Han Z. (2017). Validating Candidate Congenital Heart Disease Genes in Drosophila. Bio-Protocol.

