# Supplementary figures and images for: H3K36 Di-Methylation Marks, Mediated by Ash1 in Complex with Caf1-55 and MRG15, Are Required during Drosophila Heart Development
Source: J Cardiovasc Dev Dis. 2023 Jul 18;10(7):307. doi: 10.3390/jcdd10070307 (PMC10380788; doi:10.3390/jcdd10070307)

Supplemental Figure 1

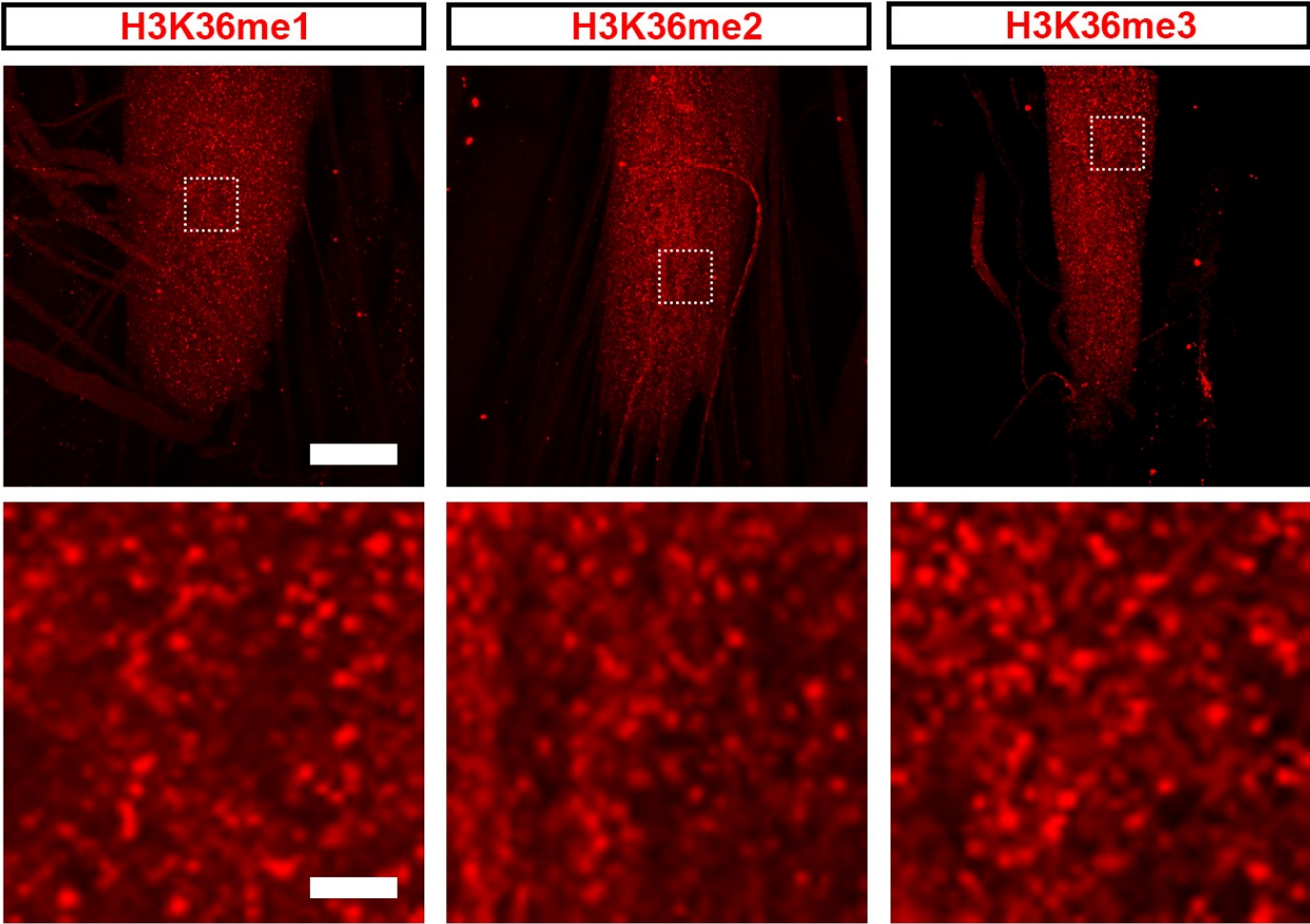

Supplement: Supplementary file 1 [file jcdd-10-00307-s001.zip › jcdd-2455233-supplementary.pdf]
